# Chloroquine Is Grossly Under Dosed in Young Children with Malaria: Implications for Drug Resistance

**DOI:** 10.1371/journal.pone.0086801

**Published:** 2014-01-23

**Authors:** Johan Ursing, Staffan Eksborg, Lars Rombo, Yngve Bergqvist, Daniel Blessborn, Amabelia Rodrigues, Poul-Erik Kofoed

**Affiliations:** 1 Projecto de Saúde de Bandim, Indepth Network, Bissau, Guinea-Bissau; 2 Malaria Research Laboratory, Department of Medicine, Karolinska University Hospital, Karolinska Institutet, Stockholm, Sweden; 3 Department of Women’s and Children’s Health, Childhood Cancer Research Unit, Karolinska Institutet, Stockholm, Sweden; 4 Centre for Clinical Research, Sörmland, Uppsala University, Sweden; 5 Dalarna University College, Borlänge, Sweden; 6 Mahidol Oxford Research Unit, Faculty of Tropical Medicine, Mahidol University, Bangkok, Thailand; 7 Centre for Tropical Medicine, Nuffield Department of Clinical Medicine, University of Oxford, Oxford, United Kingdom; 8 Department of Paediatrics, Kolding Hospital, Kolding, Denmark; Universidade Federal de Minas Gerais, Brazil

## Abstract

**Background:**

*Plasmodium falciparum* malaria is treated with 25 mg/kg of chloroquine (CQ) irrespective of age. Theoretically, CQ should be dosed according to body surface area (BSA). The effect of dosing CQ according to BSA has not been determined but doubling the dose per kg doubled the efficacy of CQ in children aged <15 years infected with *P. falciparum* carrying CQ resistance causing genes typical for Africa. The study aim was to determine the effect of age on CQ concentrations.

**Methods and Findings:**

Day 7 whole blood CQ concentrations were determined in 150 and 302 children treated with 25 and 50 mg/kg, respectively, in previously conducted clinical trials. CQ concentrations normalised for the dose taken in mg/kg of CQ decreased with decreasing age (*p*<0.001). CQ concentrations normalised for dose taken in mg/m^2^ were unaffected by age. The median CQ concentration in children aged <2 years taking 50 mg/kg and in children aged 10–14 years taking 25 mg/kg were 825 (95% confidence interval [CI] 662–988) and 758 (95% CI 640–876) nmol/l, respectively (*p* = 0.67). The median CQ concentration in children aged 10–14 taking 50 mg/kg and children aged 0–2 taking 25 mg/kg were 1521 and 549 nmol/l. Adverse events were not age/concentration dependent.

**Conclusions:**

CQ is under-dosed in children and should ideally be dosed according to BSA. Children aged <2 years need approximately double the dose per kg to attain CQ concentrations found in children aged 10–14 years. Clinical trials assessing the efficacy of CQ in Africa are typically performed in children aged <5 years. Thus the efficacy of CQ is typically assessed in children in whom CQ is under dosed. Approximately 3 fold higher drug concentrations can probably be safely given to the youngest children. As CQ resistance is concentration dependent an alternative dosing of CQ may overcome resistance in Africa.

## Introduction

Chloroquine (CQ) was the main drug for treatment of *Plasmodium falciparum* but the drug has been largely replaced due to resistance. In Africa, drug resistant *P. falciparum* disappear when CQ is no longer used and it has therefore been suggested that CQ could be re-introduced [1]–[3]. However, the rapid seasonal increase of resistant *P. falciparum* during rainy seasons and the rapid expansion of resistant *P. falciparum* throughout Africa suggest that resistance would again spread rapidly if standard doses of CQ were to be reintroduced [4], [5]. Data from Guinea-Bissau indicate that higher doses of CQ are efficacious, well tolerated and limit the spread of *P. falciparum* resistant to standard doses of CQ [6]–[11]. In the past, doses as high as 21 mg/kg daily for 3 weeks were used for treatment of amoebic liver abscess [12]. More recently, 10 mg/kg twice daily for 5 days was used for the treatment of Giardiasis and found to be well tolerated with only mild, transient and self-limited adverse events [13]. It is therefore essential to re-examine the CQ dosage regimen.

CQ was initially developed by the US army for use against *P. vivax* malaria and subsequently *P. falciparum* malaria. Initial trials were carried out on soldiers in 1946 and the dosage schedule of 25 mg/kg CQ base (total), over 3 days has changed little since then [14], [15]. However, antimalarials are mainly given to infants and children in whom renal and hepatic function, metabolic rate, extracellular and total body water, fat distribution and lean body mass change with age. Many physiological factors effecting drug disposition are better correlated to body surface area (BSA) than to body weight (BW). BSA therefore forms the basis of dose normalization with respect to variations in age, body size and body composition [16], [17]. The World Health Organization notes that CQ probably ought to be dosed according to BSA but this has never been routine practice most probably because the standard CQ dose was efficacious and well tolerated [18].

When reconsidering the dosage regimen, pharmacokinetics and toxicity must be considered. CQ is rapidly and almost completely absorbed. Peak concentrations occur 1–6 hours after oral intake and 50–65% is protein bound in plasma [19]. CQ is rapidly distributed throughout the body and accumulates in tissues including the liver, lungs, spleen and kidneys and consequently has a very large volume of distribution (>100 L/kg) [20]–[22]. CQ is metabolised in the liver and the principal metabolite is desethyl chloroquine (DCQ), which has a modest antimalarial effect [23]. When 3 mg/kg of CQ was injected intravenously, side effects were reported in all patients and there was a significant fall in systolic blood pressure and rise in heart rate which paralleled changes in plasma concentrations [22]. In line with this, serious adverse events occur soon after intake of an overdose and appear to be due to CQ’s vasodilatory effects and negative inotropism [24]–[28]. CQ is known to prolong the QT interval, and in a recent study in which the total intake of CQ was 25 mg/kg, QT prolongation was found to be dependent on CQ blood concentrations [29]. Severe adverse events thus seem to be associated with high peak concentrations that must be avoided when reconsidering the CQ dosage regimen.

The aim of this study was to describe the effect of age and body weight of children on CQ and DCQ concentrations when administered 25 or 50 mg/kg of CQ and to correlate this to the amount of CQ prescribed according to BSA.

## Methods

### Ethics Statement

Patients were included into the respective clinical studies after verbal informed consent from their caretaker. Verbal consent was obtained as literacy rates were low. A study nurse read standardized information to children and caretakers and answered questions. After approval, she signed the clinical records form to document that informed consent had been obtained. A second study nurse was present during the process. The method was approved by the ethical review board in Bissau, Guinea-Bissau. Ethical approval was granted by the ethical review board in Bissau, Guinea-Bissau (Parecer NCP/N19/2006, 019/DHE/2004 and 064/DGSP/2006), the regional ethics committee in Stockholm, Sweden (2005/111–31/1 and 2006/1151–31/1 and 2011/832–32/2) and the central scientific ethics committee in Denmark (624-01-0042). Studies 2 and 3 were also registered at ClinicalTrials.gov (study ID: PSB-2001-chl-amo and NCT00426439).

### Clinical Studies and Treatment

This report is based on 3 previous open label randomised clinical trials. The trials are published and described in detail elsewhere [6], [7], [9]. In the first [6] and second [7] studies (conducted in 1995–1996 and 2001–2004), children were randomised to observed therapy with a total dose of CQ phosphate corresponding to 25 mg/kg or 50 mg/kg CQ base. The third study was conducted between 2006 and 2008 and included children randomised to observed treatment with 50 mg/kg of CQ base [9].

### Study Site

The studies were conducted at the Bandim Health Project Demographic Health Surveillance Site (DHSS) in Bissau, Guinea-Bissau (www.bandim.org). The population is approximately 103 000 and there are 3 health centres within the DSS. Study 1 and 2 were conducted at the Bandim health centre and Study 3 was conducted at the Bandim, Cuntum and Belem health centres.

### Inclusion and Exclusion Criteria

Children aged <15 years with fever or a history of fever and with a parasite density of ≥800 *P. falciparum*/µL were included after informed consent. Children with signs or symptoms of severe malaria and children reported to have taken any antimalarial in the past week were not included.

### Treatment Schedule and Drugs

Tablets containing 160 mg CQ phosphate (corresponding to 100 mg CQ base) were used and halved when necessary. Tablets were provided by Pharmacia Upjohn, Stockholm, Sweden, for Study 1 and by Recip AB, Stockholm, Sweden, for Studies 2 and 3. Twenty five milligram per kg was given as ∼10 mg/kg on days 0 and 1 and ∼5 mg/kg on day 2. Fifty milligrams per kg was given as two daily doses of ∼10 mg/kg on days 0 and 1 and two doses of ∼5 mg/kg on day 2. Doses were repeated if a child vomited within 30 minutes of drug intake.

### Follow Up and Sampling

Children were seen twice daily on days 0, 1 and 2, and once on day 3. Starting on day 7, they were followed weekly until the end of the study. On day 7, exactly 100 µL of whole blood was put on filter papers that were dried and then put inside individual sealed plastic bags. In order to avoid contamination, great care was taken to ensure that no equipment used for this purpose was ever in contact with CQ. Children were weighed prior to treatment on day 0. BSA was estimated from body weight (BW) using the Boyd self-adjusting formula (0.9669^(BW*((0.7207–0.0003467)*(BW)))^) as this is the most accurate formula for estimating BSA [30].

### CQ and DCQ Concentration Analyses

Concentrations of CQ and its metabolite DCQ were determined by HPLC as previously described [31]. The intra-assay precision for all analytes was <5% at 2000 nmol/L, <7% at 750 nmol/L and <10% at 100 nmol/L. The inter-assay precision was <4% at 2000 nmol/L, <7% at 750 nmol/L and <12% at 100 nmol/L with a lower limit of quantitation of 100 nmol/L for all analytes.

### Statistics

Correlations were established by the Spearman rank correlation test. Median CQ concentrations and interquartile ranges were determined and compared using Quantile regression with bootstrapping (100 repetitions). Changes in BW and age of children between studies and over time were compared using the non parametric test for trend.

## Results

### Baseline Characteristics

**Figure 1:**
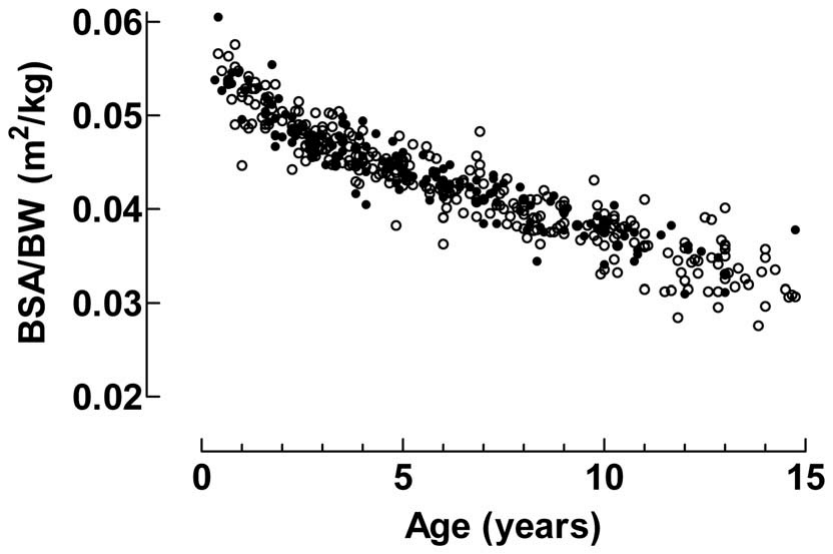
Body surface area/Body weight and age. Data from patients treated with 25/kg of CQ are given by closed circles (•) and data from patients treated with 50 mg/kg of CQ are given by open circles (○).

CQ concentrations were determined in 150 children treated with 25 mg/kg and 302 children treated with 50 mg/kg. The number of samples, age, sex, BW, median CQ and DCQ concentrations from each study are shown in Table 1. Drug concentrations were not determined if children had early treatment failure, were lost to follow up, did not take the full dose or if filter-papers with blood had been lost. In addition, filter-papers were not available at the start of the study 2 due to delivery problems. There was a significant increase of BW and age of children with year the clinical study was done (p<0.001) that was probably due to decreasing prevalence of malaria in Bissau (Table 1). The quotient BSA/BW (m^2^/kg) increased with decreasing age of the patients, Figure 1.

**Table 1:** Median age, weight and sex distribution in the different clinical studies.

Study period	1994–1995		2002–2004		2006–2008
**Dose chloroquine (mg/kg)**	25	50	25	50	50
**No of samples**	54	52	100	88	162
**Age**	4 (2–7)	4 (2–8)	6 (4–8)	5 (3–9)	7 (4–10)
**Sex (male:female)**	25∶29	22∶30	43∶57	44∶44	90∶72
**Weight (kg)**	14 (11–20)	14 (11–21)	17 (13–22)	17 (12–24)	20 (14–29)
**CQ concentration (nmol/l)**	551 (437–874)	1383 (999–1931)	546 (412–873)	1208 (822–1648)	1392 (957–2093)
**DCQ concentration (nmol/l)**	–	–	357 (238–543)	691 (327–920)	811 (549–1284)

Inter quartile range is given in brackets.

There was a significant increase in the median age and weight of children over time (p<0.001).

### CQ Concentrations

**Figure 2:**
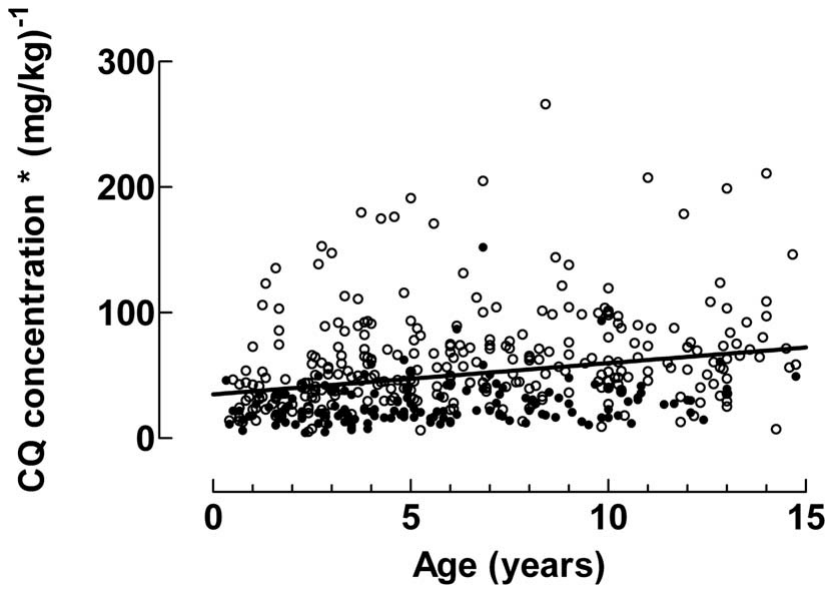
Chloroquine concentrations normalised for mg/kg. The dose normalised CQ concentrations increases with increasing age (p<0.001). The solid line is given by linear regression.

**Figure 3:**
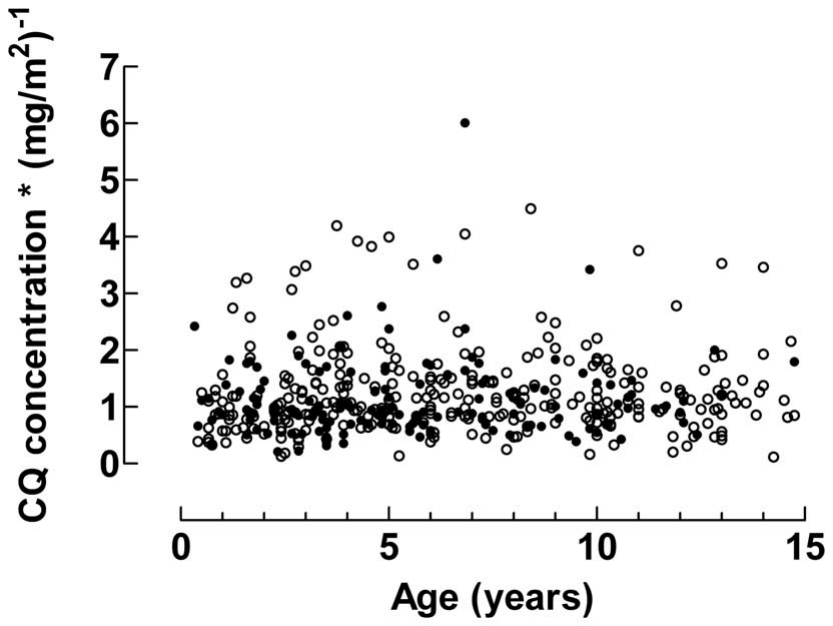
Chloroquine concentrations normalised for mg/m^2^. The dose normalised CQ concentrations were independent of age. The solid line gives the median value of CQ concentrations normalised for mg/m^2^. Data from patients treated with 25 mg/kg of CQ are given by closed circles (•) and data from patients treated with 50 mg/kg of CQ are given by open circles (○).

Median drug concentrations after intake of 25 or 50 mg/kg in children stratified by age are shown in Table 2. Irrespective of whether 25 or 50 mg/kg was prescribed, CQ concentrations decreased with decreasing age. This is further outlined in Figure 2 which shows how the CQ concentrations normalised for the dose taken in mg/kg decreased with decreasing age (slope = 2.49 [95% CI 1.60–3.38], p<0.001). In contrast, CQ concentrations normalised for dose taken in mg/m^2^ were unaffected by patient age (slope = 0.01 [95% CI, −0.01–0.03], p = 0.4), Figure 3. When the 25 mg/kg and 50 mg/kg groups were analysed separately similar results were obtained (data not shown).

**Table 2:** Median and interquartile range chloroquine concentrations (nmol/l) in different age groups.

CQ dose prescribed	25 mg/kg		50 mg/kg	
Age (years)	n	CQ concentration	N	CQ concentration
		Median (interquartile range)		Median (interquartile range)
**<2**	22	545 (392–648)	32	825 (608–1174)
**2–3**	33	462 (298–595)	61	1244 (917–1650)
**4–5**	32	540 (425–802)	40	1259 (998–1691)
**6–7**	25	704 (438–1002)	44	1517 (1102–1205)
**8–9**	16	692 (481–805)	35	1565 (1129–2469)
**10–14**	22	758 (513–944)	73	1608 (1213–2199)

CQ concentrations increased with age following intake of 25 (p<0.001) and 50 mg/kg (p<0.001). CQ concentrations in children aged <2 years prescribed 50 mg/kg were not significantly different to CQ concentrations in children 10–14 years prescribed 25 mg/kg (p = 0.67).

### Effect of Doubling the CQ Dose Per Kg in Different Age Groups

Doubling the dose of CQ taken per kg approximately doubled the measured concentration for any given BW category (Table 2). However, concentrations were not significantly different in children aged <2 years after intake of 50 mg/kg compared to children aged >10 years taking 25 mg/kg (p = 0.67).

### DCQ Concentrations

**Figure 4:**
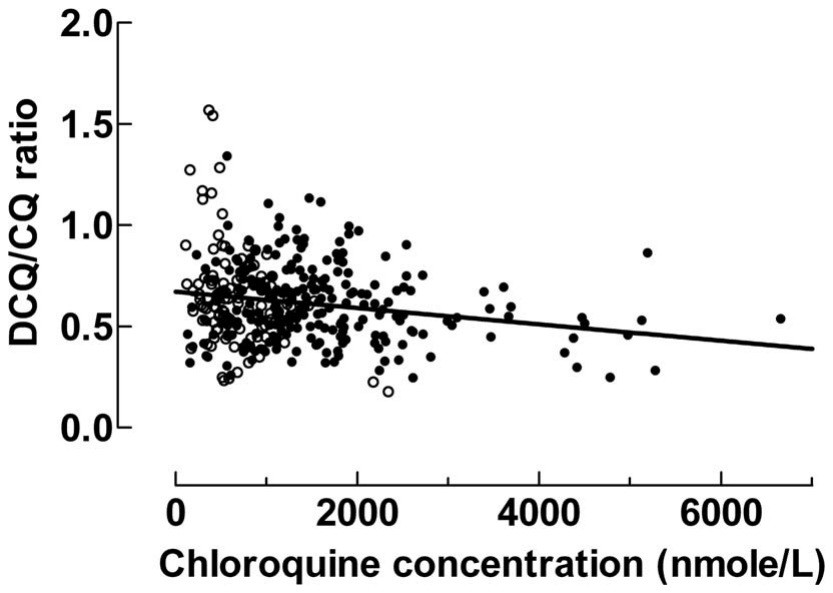
Desethyl chloroquine/chloroquine ratio as a function of chloroquine concentration. The DCQ/CQ ratio decreased with increasing CQ concentrations (p<0.001), Data from patients treated with 25 mg/kg of CQ are given by closed circles (•) and data from patients treated with 50 mg/kg of CQ are given by closed circles (•) open circles (○).

DCQ concentrations were determined in studies 2 and 3 and therefore only in 100 and 243 patients treated with 25 and 50 mg/kg respectively (Table 1). The median DCQ/CQ ratios were 0.63 (IQR 0.51–0.74) and 0.57 (IQR 0.47–0.72) after intake of 25 and 50 mg/kg of CQ, respectively (p = 0.17). DCQ/CQ ratios decreased with increasing CQ concentrations (p<0.001), Figure 4. Median DCQ and CQ+DCQ concentrations after intake of 25 or 50 mg/kg in children stratified by age are shown in supplementary materials (Table S1).

### Outliers

After intake of 50 mg/kg 11 children, with a median age of 7 years, had CQ concentrations over 4000 nmol/L without adverse events. The median DCQ/CQ ratios of 0.46 in these children was lower than the median for children taking 50 mg/kg (p<0.007). After intake of 25 mg/kg, three children had concentrations above 2000 nmol/L. For the two in whom DCQ was measured, the ratios were 0.18 and 0.23 and their ages were 10 and 6 years, respectively.

The DCQ/CQ ratio was >1 in 8/100 and 5/243 children after intake of 25 and 50 mg/kg, respectively. In these children, the median CQ concentrations were 381 and 1144 nmol/L and their median ages were 5 and 10 years.

### Adverse Events

Studies 1 and 2 compared treatment with 25 and 50 mg/kg of CQ. Both dosage regimens were well tolerated with no significant differences in adverse events. Study 3 compared 50 mg/kg of CQ with artemether-lumefantrine and the assessed clinical condition improved equally fast irrespective of treatment. Specific symptoms were sought by questioning but only pruritus was found to differ consistently between 50 mg/kg of CQ and artemether-lumefantrine treatment arms. Pruritus was found in significantly more children taking CQ than artemether-lumefantrine on days 1, 2 and 3 with 7/174 children reporting pruritus on day 3 (p<0.01). Age, CQ and DCQ concentrations were not associated with adverse events.

## Discussion

Dosing of CQ per kg resulted in significantly lower concentrations with lower age, irrespective of whether 25 or 50 mg/kg was taken. When drugs are dosed according to BW, different body composition and maturity of liver and kidney functions of infants and young children are not taken into account. For these reasons, dosing according to BSA should be preferred. When CQ concentrations were compared after adjusting for the dose given according to BSA the age related concentration differences disappeared. Thus the cause of the lower CQ concentrations was most probably insufficient dosing as indicated in a previous study and by an allometric scaling model [32], [33]. Lower concentrations can thus be avoided by dosing according to BSA rather than BW.

The currently used CQ dosing regimen has changed little since it was first determined in soldiers in the 1940s [14], [15]. The CQ concentrations found in children aged 10–14 years (758 nmol/l) are therefore likely to be closer to the originally assessed concentrations than the concentrations found in children aged <2 years (545 nmol/l) after intake of 25 mg/kg. This indicates that younger children have been and still are under-dosed. Our data indicate that children under 2 years of age should be treated with approximately double the dose per kg given to children aged 10–14 years in order to attain approximately the same whole blood CQ concentration. In accordance with this finding, a prescribed dose of 25 mg/kg would correspond to approximate dosages of 921, 770 and 510 mg/m^2^ in adults, a 12 year old child and a 2 year old, respectively (with BSAs of 1.9, 1.3 and 0.49, respectively). Thus, CQ concentrations in children aged 10–14 were probably also lower than those originally attained in adult soldiers. In line with this, the mean day 7 CQ concentration was approximately 950 nmol/l in 15 nonpregnant women after treatment of *P.vivax* with 25 mg/kg [34]. This is highly significant as treatment of children aged <15 years with 50 mg/kg of CQ successfully eradicated ∼87% of *P. falciparum* with genotypes that cause resistance to 25 mg/kg [9]. The results have major implications for the assessment of CQ efficacy in Africa as CQ resistance has typically been linked to the same genetic changes throughout the continent and because efficacy has generally been determined in children less than 5 years of age.

CQ remains the drug of choice for treatment of *P. vivax* infections and the pharmacokinetics of people with uncomplicated *P. falciparum* and *P. vivax* infections are likely to be similar. Thus children with *P. vivax* Malaria that are treated with CQ are likely to attain lower CQ concentrations compared to adults. WHO guidelines state that recurring parasitaemia before day 28 is indicative of CQ resistance. This is based on the assumption that CQ concentrations will be >100 ng/ml on day 28 after full compliance to the 25 mg/kg dose. Our data indicate that this will overestimate treatment failures in children and that time between treatment and relapse is suboptimal for identifying resistant *P. vivax* in children when the current dosing regimen is used [18], [35]. Furthermore, lower concentrations may increase the risk of treatment failure in children as well as the risk of CQ resistant *P. vivax* developing. In light of the development of CQ resistant *P. vivax* optimizing the dose given is of importance.

The ratio of DCQ to CQ concentration was found to decrease at high concentrations of CQ (Figure 4). The most probable explanation is that metabolism of CQ to DCQ was saturated at high CQ concentrations. If so, decreased metabolism might contribute to even higher drug concentrations. However, CQ’s volume of distribution is very large (>100 L/kg) and CQ concentrations are therefore primarily determined by distribution rather than metabolism suggesting that metabolism is unlikely to have a major impact on CQ concentrations during treatment [21], [36].

Eleven children had CQ concentrations over 4000 nmol/L after intake of 50 mg/kg and 3 had concentrations >2000 nmol/L after intake of 25 mg/kg. These children had low DCQ/CQ ratios but there was no correlation with BW or age. There were also a number of children with DCQ/CQ ratios >1. This was more commonly seen in children taking 25 mg/kg. CQ is primarily metabolised to DCQ via cytochrome P450 isoform 2C8 and 3A4/5 and it is possible that variations in these or other enzymes may affect the speed at which CQ is metabolised accounting for these variations [37]. Given that DCQ has a longer terminal half-life than CQ, another possible contributing factor could be intake of CQ prior to this study resulting in elevated DCQ concentrations due to its longer terminal half-life [29], [38]. An alternative explanation for the high CQ concentration with low DCQ concentration is contamination with CQ, however contamination cannot account for the high DCQ proportions.

Risk of toxicity is the main argument against increased CQ dosing. CQ is generally well tolerated but can be severely toxic causing death soon after ingestion of an overdose [24], [25]. Both 50 mg/kg and 25 mg/kg of CQ were well tolerated when given to children under 15 years of age [6], [7]. When 50 mg/kg of CQ was compared with artemether-lumefantrine, symptoms resolved equally rapidly and pruritus was the only consistent difference found. Thus 50 mg/kg is well tolerated in children <15 years of age when given as split daily doses over 3 days. Even approximately 75 mg/kg given as split doses over 5 days are well tolerated when routinely used in Guinea-Bissau as was 100 mg/kg over 5 days when used in Cuba [8], [13], [39]. Coupled with the 3 fold higher CQ concentration found in children 10–14 years old taking 50 mg/kg compared to children aged <2 years taking 25 mg/kg this indicates that young children are likely to tolerate considerably higher doses per kg bodyweight than the recommended 25 mg/kg.

Adverse events when 3 mg/kg of CQ was given intravenously over 10 minutes were correlated to peak plasma concentrations that ranged from 2454–20811 nmol/L [22]. Whole blood concentrations are approximately 10 times higher than plasma concentrations after intake of a single oral dose [40]. The day 7 concentrations found here therefore appear to be well below the concentrations causing adverse events above. More relevantly, modelling of the 25 and 50 mg/kg doses given suggests that whole blood peak concentrations occur after the 2^nd^ and 4^th^ dose respectively and are approximately 4500 and 6000 nmol/L (data not shown). This suggests that the peak drug concentrations attained with 50 mg/kg are well below those causing adverse events in line with the tolerability of higher doses discussed above. The use of split daily doses are no doubt very important as split doses enables CQ to distribute into other tissues whereby toxic peak concentrations are avoided. However, CQ concentrations show large inter-individual variation.

CQ and other quinoline antimalarials can cause prolongation of the QT time by inhibition of the rapidly acting delayed K^+^ rectifier current in myocytes [41], [42]. Inhibition of the rectifier current has been shown to be dose dependent in vitro. Furthermore, QT time corrected using Bazett’s formula increased in a CQ concentration dependent manner in healthy adult volunteers taking 25 mg/kg of CQ total dose over 3 days. Specifically QTc increased from 396 ms to 424 ms 4–5 hours after the 2^nd^ dose corresponding to the peak concentration [29]. In view of this it is perhaps surprising that the total dose of 25 mg/kg has been found to be safe through extensive use. Furthermore, a recent study indicated that treatment with CQ protected against cardiac arrhythmias in patients with systemic lupus errythematosus [41], [43]. Nontheless increased QT time is a risk factor for development of Torsade de points and is thus a major concern when higher does are used. If CQ is to be reintroduced, this ought to be preceded by thorough pharmacokinetic and pharmacodynamic studies in young children using higher doses than previously [2]. We are therefore currently undertaking a pharmacokinetic analysis of the 50 mg/kg dose and an alternative 70 mg/kg total dose (given as divided doses over 5 days) in a study that includes assessment of the cardiac effects. To date both treatment regimens have been well tolerated. Treatment with multiple doses over several days are complicated but could be simplified by use of a slow-release preparation that might enable higher doses to be given once daily whilst avoiding high peak concentrations. Furthermore, since the antimalarial efficacy of CQ is dependent on how long the parasite is exposed to concentrations of CQ that are above the minimal inhibitory concentration, a slow-release preparation would match the pharmacokinetics and pharmacodynamics of CQ better than ordinary tablets [44].

To conclude, we report that CQ concentrations decrease with age after intake of the same dose per kg and suggest that this is primarily due to a relative under-dosing of young children. The difference is so great that children aged <2 years need to take approximately double the dose per kg given to children aged 10–14 years to attain the same CQ concentration. Dosing according to BSA would probably solve the problem. We also show that children aged 10–14 years receiving 50 mg/kg have CQ concentrations that are approximately 3 fold higher than those measured in children aged <2 years taking 25 mg/kg without there being an increase of adverse events. We discuss the implications of these findings for the use of higher doses of CQ, as have been administered in The Republic of Guinea-Bissau. The results highlight the importance of correct dosing of antimalarial drugs to young children.

## Supporting Information

Table S1 **Median and interquartile range whole blood desethyl chloroquine and chloroquine+desethyl chloroquine concentrations (nmol/l) in different age groups).**
(DOCX)
