# Supplementary material for: Chloroquine Is Grossly Under Dosed in Young Children with Malaria: Implications for Drug Resistance
Source: PLoS One. 2014 Jan 23;9(1):e86801. doi: 10.1371/journal.pone.0086801 (PMC3900653; doi:10.1371/journal.pone.0086801)
Supplement: Table S1 — Median and interquartile range whole blood desethyl chloroquine and chloroquine+desethyl chloroquine concentrations (nmol/l) in different age groups). (DOCX) [file pone.0086801.s001.docx]

Table S1. Median and interquartile range whole blood desethyl chloroquine and chloroquine + desethyl chloroquine concentrations (nmol/l) in different age groups

| **CQ dose prescribed** | **25 mg/kg** | | | **50 mg/kg** | | |
| --- | --- | --- | --- | --- | --- | --- |
| **Age (years)** | n | DCQ concentration | CQ+DCQ concentration | n | DCQ concentration | CQ+DCQ concentration |
|  |  | Median (inter-quartile range) | Median (inter-quartile range) |  | Median (inter-quartile range) | Median (inter-quartile range) |
| **<2** | 11 | 200 (171-413) | 668 (456-1012) | 25 | 412 (316-623) | 1145 (810-1733) |
| **2-3** | 19 | 292 (132-335) | 753 (327-908) | 46 | 634 (391-775) | 1664 (1314-2222) |
| **4-5** | 24 | 368 (281-448) | 946 (732-1111) | 36 | 751 (603-1150) | 2160 (1684-2840) |
| **6-7** | 18 | 490 (243-719) | 1424 (789-1653) | 38 | 818 (646-1118) | 2427 (1684-3161) |
| **8-9** | 12 | 345 (271-444) | 1136 (752-1243) | 31 | 994 (675-1379) | 2459 (1991-3810) |
| **10-14** | 16 | 537 (462-643) | 1338 (975-1628) | 67 | 1057 (715-1480) | 2676 (2051-3537) |

DCQ and CQ + DCQ increased with age (p<0.001) irrespective of whether 25 or 50 mg/kg was taken.
